# On-call work and health: a review

**DOI:** 10.1186/1476-069X-3-15

**Published:** 2004-12-08

**Authors:** Anne-Marie Nicol, Jackie S Botterill

**Affiliations:** 1 Centre for Health and Environment Research, University of British Columbia, 2206 East Mall, Vancouver, BC, V6T 1Z3, Canada; 2 School of Cultural and Innovation Studies, University of East London, 4–6 University Way, London, E16 2RD, UK

## Abstract

Many professions in the fields of engineering, aviation and medicine employ this form of scheduling. However, on-call work has received significantly less research attention than other work patterns such as shift work and overtime hours. This paper reviews the current body of peer-reviewed, published research conducted on the health effects of on-call work The health effects studies done in the area of on-call work are limited to mental health, job stress, sleep disturbances and personal safety. The reviewed research suggests that on-call work scheduling can pose a risk to health, although there are critical gaps in the literature.

## Background

The question of whether work hours and schedules affect people's health has been reviewed for a range of work patterns including shift work and overtime. Research in these areas indicates that shift work, and in particular night work can interrupt sleep patterns [1], aggravate existing medical conditions and increase the risk of cardiovascular, gastrointestinal, and reproductive dysfunctions [2-4].

However, the health effects of on-call work, where workers are called to work either between regular hours or during set on-call periods, has not merited as much attention. This form of work scheduling occurs in a variety of diverse occupations, for example medical technologists, doctors, ship engineers, utility workers, electrical technicians, tug boat pilots, midwives, information technologists, media personnel and junior airline pilots. For many of these professions being on-call is not an option, but rather a component of the job. This form of scheduling is often used to provide 24 hour coverage, 7 days a week, for facilities such as hospitals and laboratories, where emergencies require personnel to immediately deal with critical situations and where the volume of evening and weekend work does not necessitate full shift coverage. Having employees on-call, even if they are being paid a stipend for their call time, is often seen as less expensive for employers than providing full shift coverage during off-peak hours [5].

While on-call work scheduling may be less expensive, it is not without human costs. On-call employees must plan their lives and the lives of their families around a call schedule. This often means limiting behaviours and obliges employees to restrict their on-call time to activities that would not interfere with their ability to work. The unpredictability of the call scheduling can also generate a great deal of stress, as home life is interrupted and workers are required to "change hats" to shift to their professional roles at any time during call. These limitations and interferences present unique challenges for on-call workers that are not encountered by those working set schedules or even people with rotating shifts. It is thus not surprising that researchers have found that on-call work patterns can have a major influence on employees' lifestyles and their interactions with family members and friends [6]. However, in addition to the impact on lifestyle and relationships, on-call work patterns may impact the health of employees.

Within the limited literature that has explored on-call work, there exists some pertinent findings concerning the impact of on-call for an employee's physical and psychological health, and social relationships, which this review seeks to bring together. Specific attention has been devoted to the areas of stress, sleep, mental health and personal safety.

### Types of on-call work

The implementation of on-call schedules varies. For many occupations, workers leave their place of employment and are placed "on-call" on evenings and weekends, which means they can be called back to work during these periods. For many professions this form of scheduling is a normal component of the occupation, for example, marine pilots can spend up to 60% of their working time on-call. However, for a limited number of occupations such as airline pilots, on-call hours are reduced with seniority. Generally, but not always, employees are compensated monetarily for the period of call, usually with a stipend which is less than their hourly rate. When on-call employees are usually expected to restrict their use of alcohol and limit distance or travel time from the work-site. The on-call experience of these workers includes aspects of interruption, either of sleep or family or social life, and often includes an element of uncertainty as to the time of call or the occurrence of the call.

Other forms of on-call include work done by junior doctors during their medical training. Medical residents spend periods of time "on-call" at a hospital, where space may be provided for them to sleep. This form of on-call work is distinct because workers remain at work to undertake their call duty. During these periods, residents often put in 30–36 hour shifts with little to no sleep [7], resulting in a combination that is both a night shift and an overtime shift. Because of the intensive demands placed on medical residents during their apprenticeship, this group has received a fair amount of research attention. This has been particularly so in the 1990s as the rigors of this period in junior doctors' training has come under much scrutiny both in the UK and in the US. New working regulations have been introduced in an attempt to deal with what is considered, by many, to be harsh and unacceptable working conditions. The debate over and outcome of these interventions continues [8-10].

This review focuses on the health effects of on-call work in which an employee spends a period of time on-call outside of their workplace and/or their regular working hours. The research on medical residents has been excluded because the medical resident experience is distinctly different from that of other professions where on-call is utilized. However, research on medical residents is used to illustrate findings from other work-related areas when appropriate.

## Methods

This review explores the published literature referring to on-call work patterns and health. For the purpose of this review, the on-call period may be formal (e.g. a person is designated as being on-call for the weekend or overnight) or informal (emergency call back during a crisis). Search terms for this review included "on-call" and "work schedule tolerance". The terms "stand-by" and "night call" are used by some professions to describe on-call type work, and were also used as search parameters. This literature review was undertaken on journal articles included in databases up to December 2000. Database searches were performed on the following electronic sources:

1) OVID Databases: including Medline (1966–2000) and Current Contents (1996–2000).

2) Canadian Centre for Occupational Health and Safety Database

3) Cambridge Abstracts (Environmental Science and Pollution Management) 1981–2000.

4) PsycInfo (1989–2000).

5) Web of Science: including Science Citation Index and Social Science Citation Index (1989–2000).

A manual review of the references generated from the computer-search was also done. Articles were excluded from the review if they were not original research, were not written in English or focused on medical residents experiences with on-call work.

Two reviewers read through each of the eligible research papers independently.

## Results

In total, 24 papers met the search criteria. Eight (8) were excluded as they focused on the impact of on-call work patterns on patient's health and not on the health of workers. The remaining sixteen studies were used for this review. The results are divided into four health-related sections; 1) Stress, 2) Sleep, 3) Mental Health and 4) Personal Safety.

### On-Call Work and Stress

Of the five studies pertaining to on-call and stress uncovered in this review, all focus on the General Practitioners (GPs) as their subject. In these studies, the relationship between on-call work and stress was measured through self-report and perceived stress.

Three of the studies were part of a major UK study carried out from 1989 to 1998 [11-13]. In the early 1990s the British health care system experienced considerable financial and administrative restructuring. This large study was conducted at different points in time to determine GP's satisfaction with the changes in their workplace. GPs were randomly selected throughout Britain in 1987, 1990 and 1998 to fill out postal questionnaires. The studies yielded sample sizes of 1817, 917, and 999 respectively, representing rather low response rates of 48%, 67% and 47%. However, the authors' assessment of all three samples found that they tended to be fairly representative of the larger population of GPs in the country [13].

In the first two studies, GPs ranked working on-call at night as one of the top two most stressful aspects of their work situation [11,12]. However results from the third study in 1998 revealed that night call was no longer a major source of stress, dropping to 12^th ^in a ranking of 14 major stressors. The authors believe this reduction in the level of stress from on-call work could be explained by the introduction of GP co-operatives in the mid 1990s for the management of out-of-hours calls. This cooperative system allowed GPs to either do their own calls or share them with a cooperative formed by 10 or more doctors. The cooperatives gave GPs greater flexibility for how and where they saw their patients and how they implemented 24-hour care and appear to have successfully reduced the stress of night visits for GPs. Indeed, night visit stress went from being one of two top stressors for GPs in 1987 and 1990 to being one of the least stressful issues by 1998. The authors also posit that this "success may also explain the reported reduction by 1998 in stress attributable to disturbance of home/family life" [[13] pg. 370].

The fourth study also dealt with the changes in the British health care system, in particular the introduction of partial shifts to decrease long on-call periods [14]. A small sample of GPs'(n = 14 and 12) were surveyed about their stress levels before and after the new system was in place. Doctors' stress levels were significantly reduced, particularly in relation to their mental well-being and their job satisfaction.

The fifth study on GPs and stress was a qualitative analysis of 25 GPs and their spouses in Manchester [15]. This research found that for male GPs, the uncertainty of being on-call caused them to be unhappy. Some doctors spoke quite frankly about how night calls could "perturb family life and wreck personal intimacy" [[15] p. 158). The uncertainty of their on-call commitments also contributed to the male GPs' unhappiness. Female GPs were stressed by factors other than on-call, including time pressure, role conflict and work overload. They were also concerned about how their work schedule decreased the amount of time they spent with their children. These marked differences between how male and female doctors experience the stress of on-call work signals the importance of examining gender as a variable in this research.

Other studies have revealed that the amount of time spent on-call varies between male and female doctors, but no clear pattern has emerged [16,17]. It has been hypothesized that female doctors who work reduced on-call hours do so because of the dual role they must play as both worker and care-giver [17,18].

Research conducted in other professions support the idea that work patterns, particularly night shifts, can increase stress in workers and have a negative impact on family life. Working late afternoon and evening shifts has been related to increased stress for both workers and their families [2]. Variable shifts have been shown to cause more stress than regular shifts [19] and working more than 50 hours per week is associated with increased job stress [20]. Many on-call workers regularly experience variation in their work patterns, as well as being expected to work at night, and undertake greater than normal hours when called in.

### On-Call Work and Sleep

Besides stress, the interruption of sleep is another major component of on-call work, particularly for those who work nights on-call and in professions that deal with emergencies that occur at all hours. Three studies have dealt specifically with the sleeping patterns and problems experienced by train and ship engineers and transplant coordinators, all of whom regularly work on-call.

The first study researched the on-call sleep patterns of 198 train engineers using prospective activity logs over a 14-day period in the United States [21]. It was determined that those working on-call had greater difficulty falling asleep and staying asleep while on-call versus when they were not on-call. Train engineers working on-call also had a greater number of days where there was less than 24 hours between the on-set of their work shifts. These engineers reported more sleep-related problems that those with at least 24 hours between the on-set of their shifts. The researchers also explored how sleeping was impacted when it was undertaken in different locations. They found that train engineers sleep varied when at home versus "away". (Engineers can finish a shift away from home, and have "away" terminals where they can sleep.) The researchers compared the amount and quality of sleep engineers had while both "at home" and "away" and found that engineers on-call slept less at home than they did "away". The authors attribute the difference to the presence of family and social obligations in the home that conflicted with the workers' ability to sleep while working on-call. However, the authors note that the response rate of this study was low, only 25% of the sampled population of approximately 800. The authors caution readers to remain critical of their findings, because their sample may be biased towards those who generally have difficulty sleeping. An analysis of the final study group did find that the responding sample reflected the age and gender distributions of the larger population, factors that the authors suggest indicate robustness even with the low response rate.

The second study of on-call and sleep explored the sleeping patterns of 53 predominantly female organ transplant coordinators in the UK, using a postal questionnaire [22]. This research determined that not only was sleep affected when people worked on-call (51% occasionally had difficulty and 6% frequently had difficulty falling asleep) but that the effects carried over to time off call as well. Sixty-eight percent of the sample reported that the time they spent on-call negatively influenced their off-call lives. Workers pointed out that after being on-call they often had to spend additional time catching up on sleep. They also complained that on-call work left them too tired to undertake social and home activities. But although the workers complained about being fatigued at home, this was not correlated with days absent from work. The authors suggest that this finding may be the result of transplant coordinators "guilt" around placing an extra burden on a co-worker if they were absent. Another possible explanation was the overall satisfaction of the type of work being done by the coordinators, a factor which may decrease their willingness to take time off.

The third study, conducted on a small sample (n = 5) of ship engineers in Sweden, measured sleep during on-call periods using electroencephalogram (EEG) and electrocardiogram (ECG) recordings and subjective ratings. [23]. This research found, like the others, that the sleep quality and quantity of the ship engineers was affected by the interruptions of being on-call. In their subjective assessments, the engineers reported being more drowsy during the day after being on-call, a finding similar to that of the transplant coordinators. But, the authors also found that the apprehension associated with the possibility of being awakened for call duty also negatively impacted sleep. On-call sleep registered less slow wave sleep (SWS) and rapid eye movement (REM) and a higher heart rate than when workers were testing during their normal sleep. Many of these conditions occurred prior to being awakened for call duty. Earlier research by the same authors examined the sleep patterns of Swedish merchant marines at sea. This population also found it difficult to fall asleep on nights when they were on watch. The anticipation of alarms that would wake them up was seen as an obstacle that prevented workers from relaxing enough to allow for normal sleep patterns to develop [24].

The impacts of sleep loss on job performance remain unclear and controversial. For example, research on the cognitive performance in sleep deprived medical residents has produced mixed results [25-27]. However, research on anaesthetists found that 86% reported fatigue related errors [28]. Job performance and fatigue have also been studied in relation to age, a factor not explored in the on-call studies. Significant changes were found between younger and older shift workers, with younger workers better able to maintain performance across day and night shifts and older shift workers prone to more sleep disruption [29].

Work-related fatigue has been related to an increase in car accidents. A review of traffic accidents determined that falling asleep while driving accounted for a major proportion of accidents while driving under monotonous conditions [30]. This finding has been corroborated with research done on medical residents working long night shifts. Seventy-five percent of accidents incurred by a population of emergency medicine residents happened after working a night shift [31]. In this study, the number of motor vehicle accidents and near misses was positively correlated to the number of nights worked per month. A similar study done on paediatric residents indicated that residents fell asleep at the wheel significantly more than other professionals, with 90% of these events occurring after a night on-call [32].

### On Call Work and Mental Health

Six studies were found that examined the impact of on-call work schedules on mental health. All of these studies used self-reported questionnaires and/or mood diaries. Five studies were conducted on GPs in the UK and one examined gas and electrical employees in France.

Two surveys were conducted by Chambers et al. [33,34] on GPs in Staffordshire, UK. The first survey, conducted in 1994 (n = 704), was designed to research the factors predictive of anxiety and depression in GPs [33]. The study determined that working one or more nights on-call per week was significantly predictive of anxiety. Other factors predictive of anxiety were depression and three or more weekdays feeling exhausted or stressed. Males and females showed no significant differences in anxiety or depression determinants.

The second survey conducted by Chambers et al in 1996 (n = 620) employed the Hospital Anxiety and Depression scale to assess the mental health of GPs [34]. It was determined that both anxiety and depression were associated with the amount of on-call duties undertaken. Findings revealed that both anxiety and depression increased with the frequency of time spent on-call per month. Again, the results were the same for both male and female GPs, and the authors conclude that GPs' mental ill health is associated with workload, of which on-call is a major factor.

A third survey done on GPs in Leeds in 1993 was designed to determine the psychological symptoms and sources of stress among 268 GPs [35]. This survey used the UK General Health Questionnaire as well as qualitative questions regarding mental health and workload. Problems with physical and mental health were significantly associated with several aspects of workload, including the amount of time spent on-call per month. The study also found that those GPs who spent more time on-call each month were more likely to feel their work affected their physical health. Males and females reported differences in the sources of their stress, with females showing greater job satisfaction than males. The authors suggest that this finding may be due to the fact that, for this study population, female doctors worked fewer hours and spent significantly fewer nights on-call [34].

The fourth study in this area surveyed mental health and job stress on 414 GPs in England in 1992 [36]. This research determined that interruptions, a category which included taking night calls, remaining alert on-call, 24 hour patient responsibility and telephone interruption of family life, was a predictive factor for decreased mental health, depression and somatic anxiety. These factors were similar for men and women, although their contribution to each condition varied by gender.

A pilot study of 44 male and female volunteer GPs using cognitive behavioural diaries assessed self-reported emotional states recorded in conjunction with hourly activities over 2 days [37]. Doctors' moods were significantly lowered when on-call as compared to off-call. Doctors on-call also had significantly increased tension and frustration. The main reported cause of dissatisfaction was the uncertain nature of the doctors working hours [37].

The sixth study examined male gas and electrical employees working in France [38]. Employees who worked on-call (n = 145) were assessed for health status and psychological problems and were compared to those not working on-call (n = 195). Workers were also questioned about the impact of their job on their family life. Although no particular mental or health disorder was found to be more frequent in the on-call group, the psychological equilibrium of the on-call workers was significantly worse than the comparison group. On-call workers also reported significantly worse global-well being and indicated significantly higher levels of social disturbance. On-call workers reported that their family and social life were acutely disturbed and they were significantly less likely to be involved in clubs or take on outside responsibilities.

The research conducted on GPs in the UK supports a negative role of on-call work related to mental health. However, the results from the gas and electrical workers do not reflect the same findings from the research on GPs. This may be the result of either a difference in study methodology or a difference that is profession-specific. On-call gas and electrical workers did experience psychological disruption and the lack of significant diagnostic findings may be a function of other factors, such as self-selection, in this profession, where those most affected opt out early on. The on-call gas and electrical workers experience of family and social life disruption does mirror the experiences of doctors and transplant coordinators as discussed previously [13,15,22].

### On-call Work and Personal Security

Working on-call often necessitates leaving home alone, at night, to attend work, conditions that can jeopardize personal safety. Unfortunately, there is only sparse data regarding this issue. A study done in the north west of England, in a hospital where the on-call sleeping quarters were separate from the hospital found that 40% of anaesthetists feared for their safety while walking through hospital grounds at night [39].

In medical professions, patients can also present a danger to those working on-call. Doctors have cited fear of violence from night call visits as a significant stressor [11]. A study of 327 nurses in remote areas who worked on-call found increased incidences of violent acts perpetrated by patients, particularly in smaller communities [40]. This study found that working on-call increased the number of incidents ranging from verbal abuse to property crime and physical assault compared to working regular shifts.

This issue has only been peripherally studied and further attention needs to be given to personal safety, particularly when being called in at night.

## Discussion

What emerges from this review is the limited research that has been done in the area of on-call work. Preliminary work done in the areas of stress and mental health suggests that on-call work may play a role in increasing stress and decreasing mental well-being. The three studies that examined sleep indicate that on-call work does decrease the quality and quantity of sleep for workers and can leave people feeling fatigued for periods after their on-call work.

The current body of literature on the health effects of on-call work is limited in part due to the narrow range of professions studied. The majority of research done to date has been on general practitioners. It is reasonable to assume that the effects of on-call will vary across occupations, given the host of other factors that can influence occupational health. However, the degree to which this variation exists might only be determined by examining a wider occupational base. The need to undertake more on-call research across a greater variety of occupational groups is suggested given that this form of work scheduling touches many occupations, and given that on-call work is estimated to continue to increase in many sectors in the future [6].

There is also an obvious lack of research focusing on the impact of on-call shifts on psychosocial factors. Given the very disruptive and limiting nature of on-call schedules, it would not be surprising that workers' family and social life suffer due to this type of scheduling. The results of the research addressing gender (discussed above) do suggest, albeit indirectly, that such social and familial impacts may be significant. However, without more research, it is not possible to determine the magnitude of these effects, nor the relative importance compared to other factors such as physiological responses.

More rigorous methodological designs are needed for future research in the area of on-call work and health. The current research is predominantly cross-sectional in nature, a factor that makes it difficult to determine causality. Only two studies employed external comparison groups [21,38] and only a limited number have measured effects in workers on-call versus off-call (own-controls) [22,23]. Additionally, most of the measurement has been subjective in nature and often the operationalization of on-call work is not clear. In the GP studies, on-call is generally measured as the "number of nights spent on-call" either per week or per month. Some attempt is made to measure the amount of sleep during these periods, but there is little refinement of factors such as whether the subject were actually called in to work and for how long. Additionally, little attention has been paid to the amount of time worked or sleep obtained prior to the on-call shifts or factors such as second jobs or outside work, variables that may confound the outcomes. Other factors, such as age and personality type, that have been shown to be significant variables in other areas of work scheduling [41,42] also need to be explored. Attention also needs to be paid to the possible self-selection of workers out of on-call professions or adaptive strategies that workers may employ to cope with on-call (such as the sharing of on-call shifts). More controlled research that includes both subjective and objective measures would provide better evidence regarding the effects of on-call work.

Future research on the health effects of on-call work also needs to examine the role of gender, not only from a physiological standpoint, (e.g. reproductive issues), but also from a psychosocial perspective. Many of the articles reviewed above indicate differences in how males and females experience the stress of on-call work [11,15,17,36]. Research in other work-related areas suggests that males and females cope differently with the impact of job schedules [43-45]. While gender may be a factor that directly mediates health effects, it may also be an indirect measure of other phenomena such as the division of labour outside of the workplace. More careful research is needed to illuminate the role gender may play in the effects of on-call work.

The range of health effects studied in relation to on-call work has to date been inadequate. Health conditions such as cardiovascular disease, reproductive problems, gastrointestinal issues and overall mortality need to be explored as has been done in conjunction with work patterns such as overtime and shift work [41,45]. Factors such as personal safety and car accidents have only briefly been touched upon, and merit more attention.

## Conclusions

While the results of this review are limited, initial research in this area suggest that being on-call can have negative impacts on workers' sleep patterns, mental health and personal life. Further research in this area is required to provide a clear picture of the risks of this form of work scheduling.

## List of abbreviations

UK, United Kingdom

US, United States of America

## Competing interests

The authors declare that they have no competing interests.

## Authors' contributions

AMN designed the research project, carried out the literature search, reviewed articles and drafted the manuscript. JSB reviewed articles and edited the manuscript. Both authors approved the final manuscript.
